# Shockingly Unreliable: A Case Report of Failure of Synchronized Cardioversion Device to Capture, Resulting in Ventricular Fibrillation and Cardiac Arrest

**DOI:** 10.5070/M5.53094

**Published:** 2026-10-31

**Authors:** Clifford J Buckley, Bradley J McClintic, Andrew L Juergens, Shea Standley

**Affiliations:** * Baylor Scott & White Medical Center, Department of Emergency Medicine, Temple, TX; ^ Baylor College of Medicine, Temple, TX

## Abstract

Synchronized cardioversion is a standard treatment for unstable ventricular tachycardia. While modern defibrillators are designed with safety mechanisms, synchronization failure can result in catastrophic complications. We present the case of a 65-year-old woman with unstable wide-complex tachycardia with a pulse who underwent emergent synchronized cardioversion. Despite activation of the “sync” mode, the device failed to capture appropriately and delivered a shock on the T-wave, precipitating ventricular fibrillation and cardiac arrest. After 26 minutes of resuscitation and multiple defibrillations, return of spontaneous circulation (ROSC) was achieved. The patient ultimately recovered and was discharged home in stable condition. This case underscores the rare but serious risk of synchronization failure, particularly in obese patients where pad placement and capture may be impaired,^1^ and emphasizes the importance of visually confirming synchronization prior to shock delivery.

**Topics:**

Cardioversion, ventricular fibrillation, defibrillator malfunction, ACLS, emergency medicine.

**Figure:**
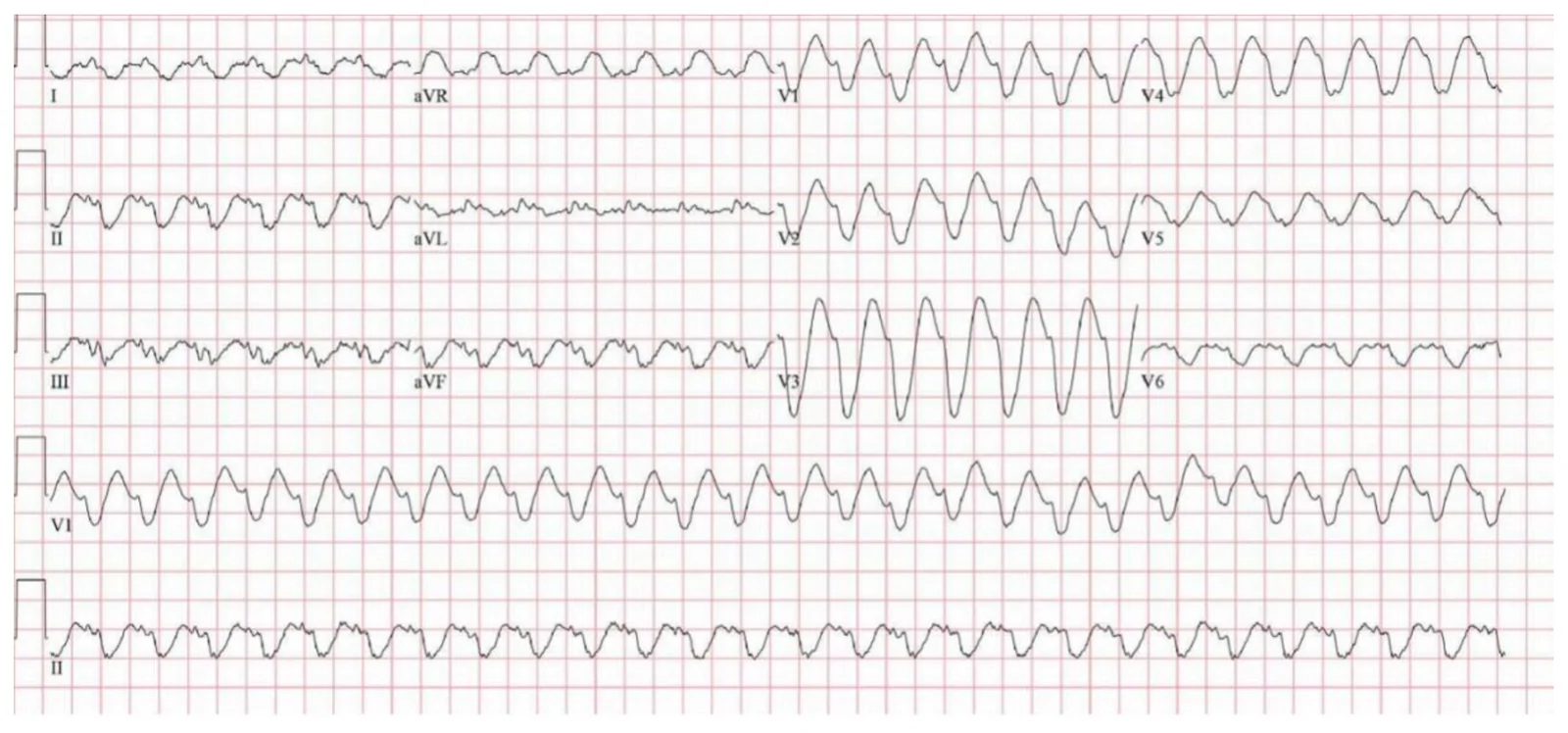
Pre-Cardioversion ECG

**Figure:**
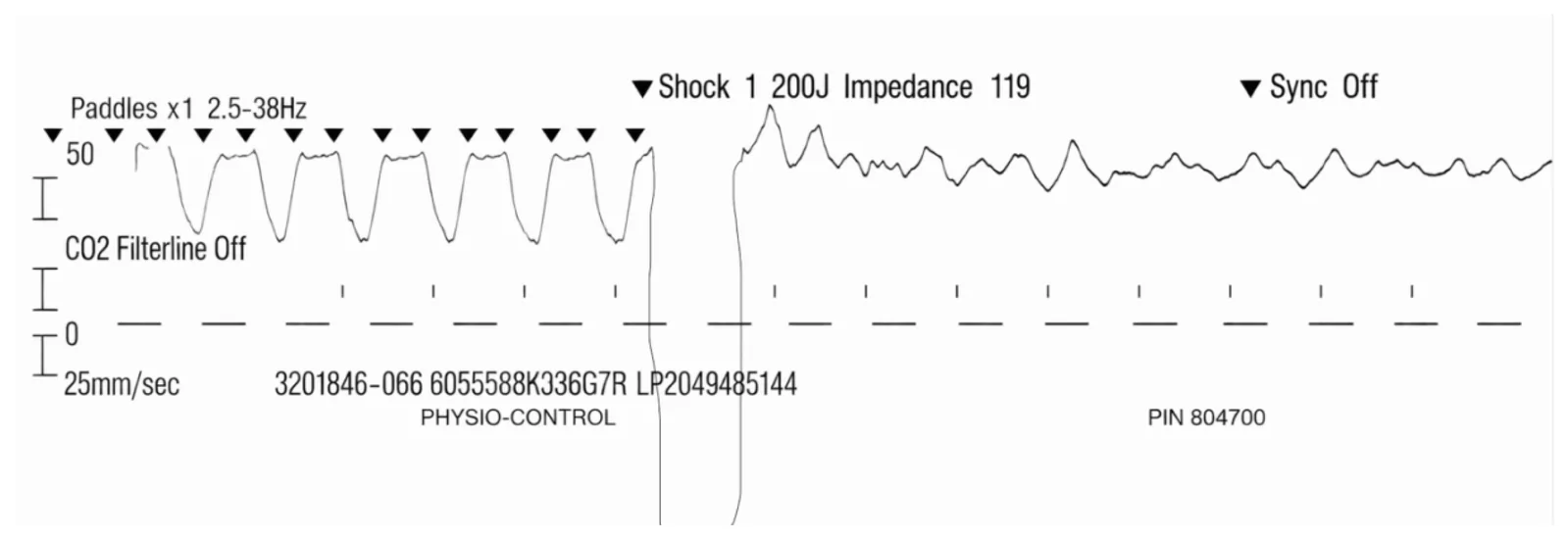
Post Cardioversion Rhythm Strip

**Figure:**
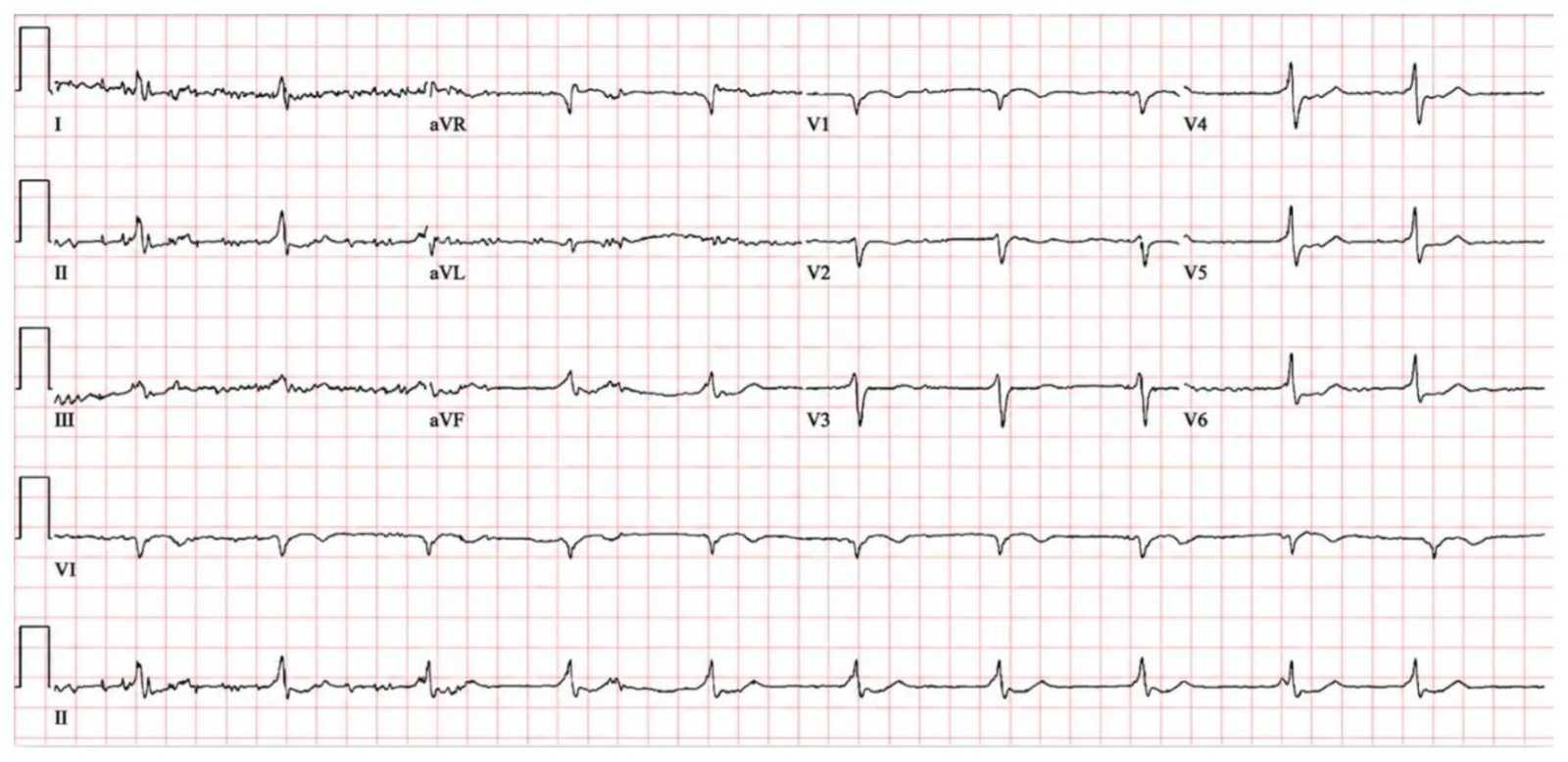
Post-ROSC ECG

## Brief introduction

Synchronized cardioversion is a commonly performed procedure in the emergency department to treat unstable ventricular tachycardia (VT). Synchronization prevents shock delivery during the vulnerable T-wave, minimizing the risk of degeneration into ventricular fibrillation (VF).^2^ There are classic teachings about the risk of so-called “R on T phenomenon” that can happen with unsynchronized cardioversion, but there is very limited literature or case reports on the subject. We report a case of synchronization failure leading to cardiac arrest, highlighting the need for clinician vigilance and optimization of technique, particularly in obese patients.

## Presenting concerns and clinical findings

A 65-year-old woman with a history of chronic obstructive pulmonary disease (COPD), heart failure with preserved ejection fraction, and paroxysmal atrial fibrillation (on flecainide and anticoagulation) presented to the emergency department via ambulance for palpitations and flushing during physical therapy. She had taken an extra 25 mg dose of her home metoprolol without relief.

## Significant findings

On arrival, she was alert and oriented. Her vital signs were heart rate (HR) ~150 beats per minute (BPM), blood pressure (BP) 130/68 mmHg, oxygen saturation within normal limits, and mild tachypnea. The initial electrocardiogram (ECG) demonstrated wide-complex tachycardia (WCT) at 150 BPM (Pre-Cardioversion ECG). She denied chest pain, dyspnea, recent illness, or medication changes.

Laboratory studies, including electrolytes, complete blood count, and troponin, were unremarkable except for a brain natriuretic peptide (BNP) of 132 pg/mL. Chest X-ray and computed tomography (CT) of the chest were pending at the time of decompensation.

She received 150 mg intravenous (IV) amiodarone for stable wide complex tachycardia in an effort to rhythm control. The patient was mildly diaphoretic but generally well-appearing with stable vitals making medical management the first line of treatment. Over the next 30 minutes, her dyspnea worsened, and her BP decreased to 98/78 mmHg. Emergent synchronized cardioversion was planned due to patient’s decompensation.

After giving her ketamine 1 mg/kg IV for sedation, adhesive defibrillator pads were placed anterior–posterior, and the device was set to “sync” mode. A shock was delivered, but immediately afterward the rhythm degenerated into ventricular fibrillation (Post-Cardioversion Rhythm Strip). In the figure, the arrows indicating the machine’s interpretation of the QRS complex can be seen double counting each QSR with the shock being delivered on the second of the two markers, which ends up being in the critical refractory period causing the patient to decompensate into pulseless ventricular fibrillation.

## Patient course

Advanced Cardiac Life Support (ACLS) was performed, including multiple unsynchronized defibrillations, magnesium sulfate, calcium gluconate, intubation, and norepinephrine initiation. After 26 minutes, ROSC was achieved. Post-ROSC ECG showed junctional rhythm with atrioventricular (AV) dissociation and no ST-elevation myocardial infarction (STEMI) criteria (Post- ROSC ECG).

Imaging returned negative for pulmonary embolism but demonstrated pleural effusions, rib fractures, and ground-glass opacities. She was admitted to the cardiovascular intensive care unit, weaned from vasopressors, diuresed, and extubated on hospital day two. She was transitioned to oral amiodarone, resumed anticoagulation, and discharged home in stable condition.

## Discussion

This case highlights a rare but critical complication of synchronized cardioversion: failure of the device to correctly sense QRS complexes and inappropriate shock delivery during the T wave, precipitating ventricular fibrillation. While the safety mechanisms in modern defibrillators are highly reliable, they are not guaranteed. In synchronized mode, the device analyzes the cardiac rhythm and times shock delivery to coincide with the R wave, avoiding the vulnerable repolarization period of the T wave^3^. If the device fails to sense properly, the shock can be delivered at an unsafe point in the cardiac cycle, triggering VF through the “R-on-T” phenomenon.

Several factors may contribute to synchronization failure. Obesity can impair accurate QRS sensing due to increased transthoracic impedance and suboptimal pad–skin contact. Inadequate adhesive pad placement, movement artifact, or low-amplitude QRS complexes can also reduce signal quality, causing delayed or absent synchronization markers.^1^ Additionally, wide-complex tachycardias with atypical morphologies may pose technical challenges for the device’s algorithms, further increasing the risk of mistimed shocks.

In our patient, anterior–posterior pad placement was chosen, but her body habitus may have contributed to poor sensing.^4^ Studies have demonstrated that applying manual pressure or using handheld paddles can improve transthoracic current delivery and enhance sensing accuracy in obese patients undergoing cardioversion.^5^ This aligns with best-practice recommendations emphasizing visual confirmation of sync markers before every shock.

Early recognition and immediate response are crucial. Clinicians should always be prepared for immediate defibrillation if synchronization fails precipitating pulseless ventricular tachycardia (VT). Protocols should emphasize rapid rhythm reassessment after each shock and clear team communication because delays in defibrillation significantly worsen survival outcomes in VF.^6^ Simulation-based team training may further improve the rapid response to rare but high-stakes equipment failures in the emergency setting.

Beyond the acute event, this case also underscores the importance of post-resuscitation care. ROSC after prolonged VF arrest requires meticulous hemodynamic optimization, targeted temperature management when indicated, and evaluation for reversible triggers. In this patient, absence of acute coronary occlusion and stable recovery highlighted a purely iatrogenic event related to synchronization error—reinforcing why procedural vigilance can be lifesaving.

In summary, although synchronization failure is rare, it can have catastrophic consequences. Optimizing pad placement, applying manual pressure in patients with challenging body habitus, and visually confirming synchronization markers are essential steps to minimize risk. If the synchronization is noted to be misaligned, potential solutions would be replacement of the pads or exchanging of the defibrillator with reassessment of the synchronization setting before delivering electricity. Emergency physicians, nurses, and advanced practitioners should maintain a high index of suspicion for this complication and be trained to respond without hesitation.

Synchronized cardioversion is a safe and effective therapy, but rare device failures can result in catastrophic arrhythmias. Clinicians should confirm synchronization visually and optimize pad technique, especially in obese patients. Awareness of this potential complication allows for faster recognition and prompt management, reducing the risk of adverse outcomes.

## Supplementary Information






